# Facet-Engineered BiVO_4_ Photocatalysts for Water Oxidation: Lifetime Gain Versus Energetic Loss

**DOI:** 10.1021/jacs.4c09219

**Published:** 2024-09-21

**Authors:** Tianhao He, Yue Zhao, Daniele Benetti, Benjamin Moss, Lei Tian, Shababa Selim, Rengui Li, Fengtao Fan, Qian Li, Xiuli Wang, Can Li, James R. Durrant

**Affiliations:** † Department of Chemistry, Centre for Processable Electronics, Imperial College London, London W12 0BZ, U.K.; ‡ State Key Laboratory of Catalysis, Dalian Institute of Chemical Physics, Chinese Academy of Sciences, Dalian National Laboratory for Clean Energy, Dalian 116023, China

## Abstract

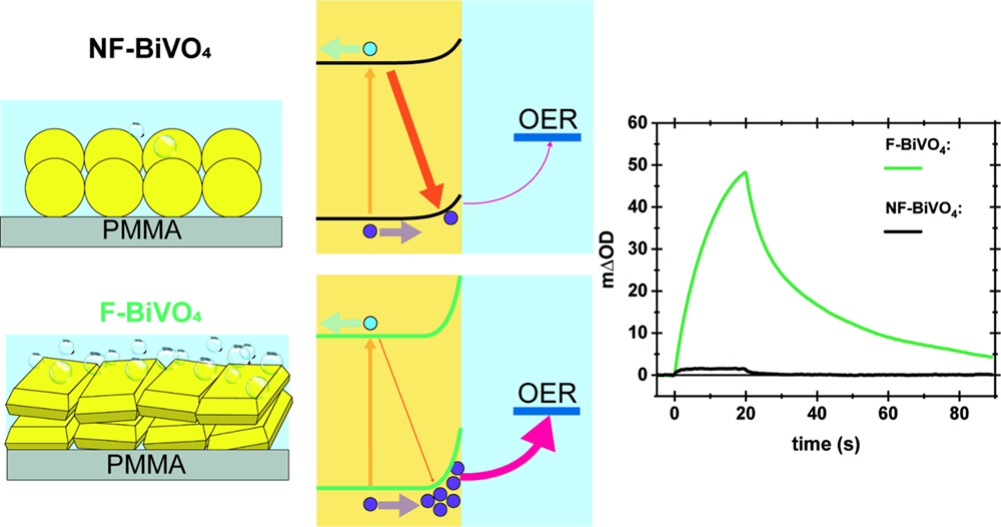

A limiting factor to the efficiency of water Oxygen Evolution Reaction (OER) in metal oxide nanoparticle photocatalysts is the rapid recombination of holes and electrons. Facet-engineering can effectively improve charge separation and, consequently, OER efficiency. However, the kinetics behind this improvement remain poorly understood. This study utilizes photoinduced absorption spectroscopy to investigate the charge yield and kinetics in facet-engineered BiVO_4_ (F-BiVO_4_) compared to a non-faceted sample (NF-BiVO_4_) under operando conditions. A significant influence of preillumination on hole accumulation is observed, linked to the saturation and, thus, passivation of deep and inactive hole traps on the BiVO_4_ surface. In DI-water, F-BiVO_4_ shows a 10-fold increase in charge accumulation (∼5 mΔOD) compared to NF-BiVO_4_ (∼0.5 mΔOD), indicating improved charge separation and stabilization. With the addition of Fe(NO_3_)_3_, an efficient electron acceptor, F-BiVO_4_ demonstrates a 30-fold increase in the accumulation of long-lived holes (∼45 mΔOD), compared to NF-BiVO_4_ (∼1.5 mΔOD) and an increased half-time, from 2 to 10 s. Based on a simple kinetic model, this increase in hole accumulation suggests that facet-engineering causes at least a 50–100 meV increase in band bending in BiVO_4_ particles, thereby stabilizing surface holes. This energetic stabilization/loss results in a retardation of OER relative to NF-BiVO_4_. This slower catalysis is, however, offset by the observed increase in density and lifetime of photoaccumulated holes. Overall, this work quantifies how surface faceting can impact the kinetics of long-lived charge accumulation on metal oxide photocatalysts, highlighting the trade-off between lifetime gain and energetic loss critical to optimizing photocatalytic efficiency.

## Introduction

Harnessing sunlight to drive the synthesis of chemical fuels and feedstocks has gained increasing attention over recent years. Among the range of approaches under investigation, the use of photocatalyst particle suspensions or sheets has been suggested to hold particular promise for low-cost sunlight conversion.^1^ However, while promising progress is being made, such photocatalytic systems exhibit efficiencies that are too low for viable commercialization.^2^ In most such systems, the primary reason for such low efficiencies is the kinetic competition between rapid charge recombination and slower interfacial charge transfer/catalytic processes. This kinetic challenge is particularly severe for photocatalysts driving overall water splitting due to the slow (ms-s) timescale of water oxidation catalysis.^3^ A further consideration is the energetic cost of lifetime gain. We have recently reported a correlation across a broad range of photovoltaic and photosynthetic solar conversion systems that a 10-fold increase in charge carrier lifetime typically comes at an energetic cost of ∼59 to 90 meV.^4^ For photoelectrochemical systems, an applied bias can be employed to drive the spatial separation of photogenerated electrons and holes and thus retard their recombination, but at an energetic cost resulting from this applied potential. In contrast, photocatalysts must achieve an equivalent charge separation without any applied bias, making this particularly challenging. A range of strategies have been proposed to drive such charge separation, including, most notably, the formation of heterojunctions, the deposition of cocatalysts (e.g., Pt, FeNiOOH, CoPi, etc.), and facet engineering.^5,6^ Since the seminal work on facet-controlled synthesis of TiO_2_,^7^ facet-engineering has proven to be a particularly effective method to enhance the efficiency in various types of materials, particularly particle-based metal oxide semiconductors.^8^ Facet-engineering involves the manipulation of nucleation and growth rates to expose facets with different surface energies selectively.^6^ However, quantitative analysis of the extent to which such facet-engineering modulates the lifetimes and energetics of photogenerated charges in such photocatalysts has, to date, received only limited study.^6,9,10^ Herein, we address this uncertainty, employing time-resolved absorption spectroscopies to quantify the impact of facet-engineering on the lifetimes and energetics of photogenerated holes in bismuth vanadate (BiVO_4_) photocatalyst particles.

Anatase TiO_2_ was the first material found to exhibit significant increases in photocatalytic performance after crystal facet-engineering to increase the exposure of particular facets.^11^ Facet-engineering has since been shown to improve the performance of SrTiO_3_,^12^ WO_3_,^13^ and BiVO_4_ photocatalysts.^14,15^ Notably, unity quantum efficiency photocatalytic water splitting has recently been reported by Domen et al. under ultraviolet irradiation with faceted Al:SrTiO_3_ photocatalyst particles. Among these materials, BiVO_4_ is of particular interest due to its narrower (2.4 eV) band gap, allowing the absorption of visible solar irradiation. As such, several studies have addressed the factors that enhance facet-driven charge separation in this material.^15,16^ Some of us have previously reported a 40-fold enhancement in photocatalytic water oxidation performance for faceted BiVO_4_ photocatalyst particles relative to a non-faceted control when using a reversible redox couple as an electron acceptor.^17^ The enhancement was correlated with evidence that photogenerated electrons predominantly accumulated on (010) facets, while holes accumulated on (110) facets. This separation of charges, induced by the facets, was attributed primarily to differences in surface energies caused by the facets’ distinct crystal terminations, which led to band bending that favors the separation of holes and electrons toward the (110) and (010) facets, respectively.

Kelvin Probe Force Microscopy (KPFM) has been employed to measure the contact potential difference (CPD) generated by electron and hole accumulation on (010) and (110) BiVO_4_ facets, respectively.^18,19^ The magnitude of band bending induced by facet-engineering was estimated to be approximately 50 mV.^17,18^ However, this value is significantly smaller than the band bending caused by applied bias in BiVO_4_ photoanodes to achieve efficient water oxidation (onset potential typically 0.3–0.5 V positive of the flat band).^20,21^ As such, it is unclear if the band bending induced by facet engineering of BiVO_4_ is sufficiently large to explain fully the enhanced photocatalytic performance. Density functional theory (DFT) analyses of BiVO_4_ have suggested that the spatial separation of electrons and holes could also arise from an asymmetry in charge carrier mobilities toward different facets.^9^ In addition, electrochemical impedance spectroscopy (EIS) studies have indicated that co-catalysts on specific exposed facets in such faceted materials can reduce the charge transfer resistance at the semiconductor/electrolyte interface.^22^ However, direct measurements of the impact of facet engineering on the charge carrier dynamics in metal oxide photocatalysts have been very limited to date. As such, while facet-engineering has been established as a very promising route to enhance photocatalytic activity, its impact on the charge separation, recombination, and catalysis dynamics, which underlie this activity, remains poorly understood.

In the study herein, we utilize photoinduced absorption spectroscopy (PIA) to directly monitor the accumulation and kinetics of photogenerated holes on facet-engineered BiVO_4_ (F-BiVO_4_) under various conditions and compare these results with those from a non-faceted BiVO_4_ (NF-BiVO_4_) control. Studies were undertaken in the absence or presence of a Fe^3+^/Fe^2+^ reversible redox couple. This redox couple has been shown to be an effective electron acceptor for F-BiVO_4_ photocatalysts, extracting electrons from the (010) facet for potential subsequent utilization in a tandem photocatalytic water-splitting system.^23^ The undesired extraction of holes by Fe^2+^ ions, which would compete with the Oxygen Evolution Reaction (OER), has been observed to be suppressed, attributed to Coulomb repulsion between positively charged (110) facets and Fe^2+^ ions.^17^ Our PIA investigation enables us to quantify the impact of facet engineering on the accumulation of long-lived photogenerated holes on faceted and non-faceted BiVO_4_ under operando conditions. We identify a substantial impact of pre-illumination upon the observed hole accumulation, attributed to the filling of deep and unreactive hole traps on the BiVO_4_ surface. Under one sun-equivalent irradiation, facet-engineered BiVO_4_ exhibits a 30-fold increase in the accumulation of long-lived holes required for water oxidation compared to the non-faceted control BiVO_4_ sample. The long lifetime of these holes indicates an energetic stabilization relative to the bulk in the order of hundreds of meV. Consistent with this observation, F-BiVO_4_ exhibited ∼5-fold slower water oxidation kinetics than non-faceted BiVO_4_ photoanodes under positive applied bias. These results provide a quantitative description of the impact of facet-engineering on charge carrier dynamics and energetics of BiVO_4_ photocatalysts.

## Results

**Figure 1:**
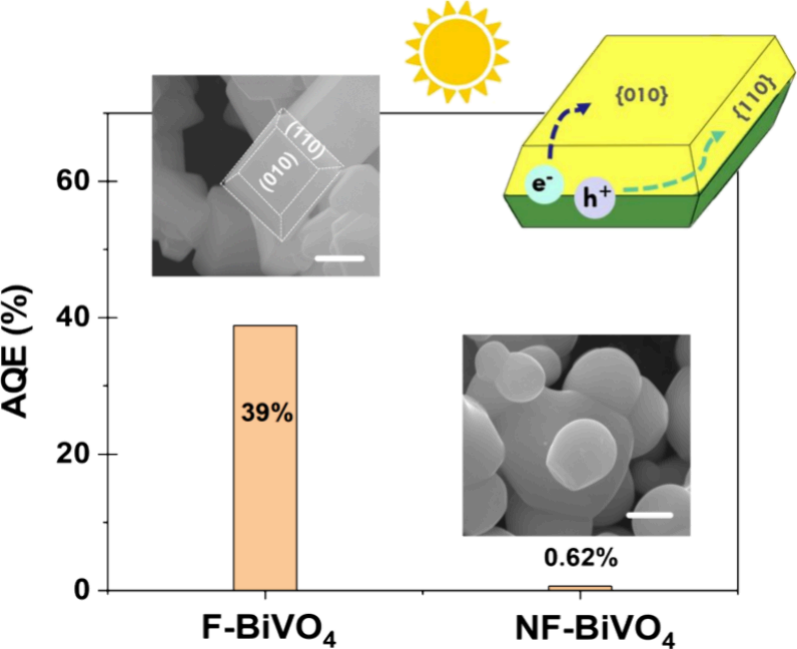
SEM images and Apparent Quantum Efficiency (AQE) for water oxidation of F-BiVO_4_ and NF-BiVO_4_ photocatalyst films in 10 mM Fe(NO_3_)_3_ solution (used as electron acceptor) under 300 W Xe light source with 420 nm band-pass filter. Scale bar in SEM images represents 500 nm. See also ref (16).

F-BiVO_4_ and NF-BiVO_4_ photocatalysts were prepared by a hydrothermal synthesis; the synthesis method, particle morphology, optical and photocatalytic performance have been previously reported.^17^ The synthesised F-BiVO_4_ and NF-BiVO_4_ possess comparable particle sizes, with diameters spanning several micrometres. However, unlike NF-BiVO_4_, F-BiVO_4_ exhibits distinct exposed (010) and (110) facets (Figure 1). Additional SEM images of the film samples are provided in the ESI (Figure S1), showing that the particles retain their morphology when deposited as film and are arranged in a random orientation. F-BiVO_4_ dispersion showed an apparent quantum efficiency (AQE) of 63% for the OER in a Fe(NO_3_)_3_ solution serving as an electron acceptor when illuminated with a 300 W xenon light source (λ ∼ 400–440 nm). This efficiency is 70 times higher than the 0.87% observed for NF-BiVO_4_ dispersion, as shown in Figure S2. However, due to the large particle size of the sample, strong stirring is required to keep the dispersion stable, which introduces significant noise into our spectroscopy measurements. Therefore, we chose to use films made from the same particles for our spectroscopy measurements. The AQE data of these films, shown in Figure 1, indicate that the water oxidation efficiency of F-BiVO_4_ (39%) is 62 times higher than that of NF-BiVO_4_ (0.62%). Although the performance of both film samples is relatively lower than that of the dispersion, this discrepancy is due to challenges with electrolyte penetration and the desorption of oxygen or hydrogen from the dense particulate photocatalyst layer. Nevertheless, the photocatalyst sheet system preserves the inherent activity of the particulate photocatalyst. Similar observations have been reported by Domen’s group.^24^ Despite the differences in performance, the relative comparison between F-BiVO_4_ and NF-BiVO_4_ is consistent with the suspension particulate data observed. Overall, these data align with values previously reported, confirming the successful preparation of F-BiVO_4_ nanoparticles.^17^

KPFM measurements were also conducted in air for these two samples, as shown in the ESI (Figure S3). The CPD data in the dark indicated a CPD difference of approximately 60 meV between the (010) and (110) facets of F-BiVO_4_. These values are consistent with those measured for similar particles in previous studies.^17^ This difference suggests the formation of a built-in electric field within the F-BiVO_4_ particles, which can enhance charge separation. In contrast, the CPD data for NF-BiVO_4_ showed no variation across the particle surface. This observation further supports the AQE differences observed in Figure 1.

To further investigate the origin of the performance enhancement arising from facet-engineering, we employed PIA spectroscopy to compare the kinetics of hole accumulation in the F-BiVO_4_ and NF-BiVO_4_ nanoparticles deposited on poly(methyl methacrylate) (PMMA) substrates (see ESI for full experimental details). PIA measurements utilize a prolonged light pulse (in the order of seconds) to drive the accumulation of long-lived charges under operando “quasi-steady state” conditions. This technique has been shown to be particularly effective at monitoring the accumulation of long-lived holes driving the kinetically slow OER in a range of metal oxide photoanodes and photocatalysts, including BiVO_4_.^25,26^ In this study, the samples were illuminated for 20 s using a 365 nm LED to induce charge accumulation for each measurement. The LED light was then switched off for 70 s to monitor the decay kinetics of the accumulated charges. Unless otherwise stated, the LED intensity was ∼12.6 mW/cm^2^, corresponding to approximately one sun irradiation. The photoinduced absorption spectra measured after 20 s irradiation for F-BiVO_4_ and NF-BiVO_4_ are reported in Figures S6 and S7, respectively. Both samples have a broad absorption with a maximum at ∼550 nm, characteristic of BiVO_4_ holes.^25,27^ The predominance of hole absorption over electron absorption is further confirmed by the suppression of this signal when a hole scavenger (methanol) is present, and an amplification of this signal upon the addition of an electron scavenger (Fe^3+^) (Figures S6 and S7). We note that photogenerated BiVO_4_ electrons have been shown to undergo rapid trapping, with the trapped electrons resulting primarily in a narrow absorption signal around 460 nm, a wavelength inaccessible in this study due to light scattering limitations.^28^ Therefore, herein, we focus on the PIA signal probed at λ = 550 nm to investigate the dynamics of photogenerated BiVO_4_ holes.

**Figure 2:**
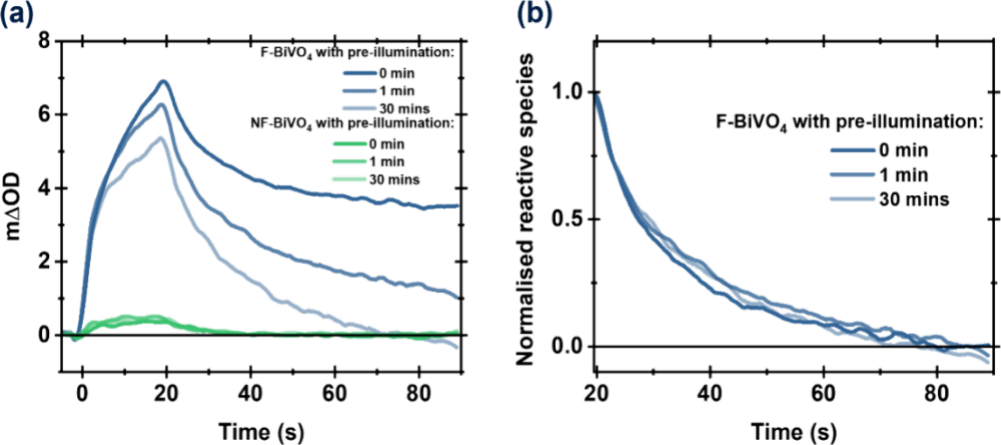
(a) PIA traces in aerobic DI-water probed at 550 nm of F-BiVO_4_ (blue) and NF-BiVO_4_ (green) following preillumination periods of 0, 1, and 30 min. (b) Normalized PIA kinetics of reactive charges in F-BiVO_4_ with 0, 1, and 30 min preillumination.

Figure 2a shows the 550 nm PIA transients of F-BiVO_4_ and NF-BiVO_4_ films in water with different pre-illumination times, measured in DI-water without an electron scavenger. The PIA signal for the F-BiVO_4_ is 10-fold larger than that of NF-BiVO_4_. Furthermore, the PIA signal for F-BiVO_4_ does not saturate during the 20 s LED irradiation, suggesting that this 10-fold increase in hole accumulation due to faceting represents a lower limit.

It is evident that faceting is sufficient to enable the accumulation of remarkably long-lived (in the order of seconds) BiVO_4_ holes even in the absence of an electron acceptor, indicating a significant facet-induced band bending driving the spatial separation of electrons and holes. Before investigating the facet-induced charge accumulation in more detail, we first consider the impact of pre-illumination on the kinetics of the accumulated holes, as shown in Figure 2. Pre-illumination was carried out using the same 365 nm, 1 sun equivalent LED used for the PIA measurements, but operated continuously for several minutes until 50 s prior to starting the pulsed PIA measurements. The PIA measurements after pre-illumination only monitor the optical changes caused by reactive holes, as the absorbance of trapped holes is already included in the baseline. Figure 2a demonstrates that pre-illumination of the F-BiVO_4_ results in a significant acceleration of the PIA signal decay, along with a slight decrease in the signal amplitude. In contrast, pre-illumination appears to have no substantial effect on NF-BiVO_4_. More specifically, the signal for F-BiVO_4_ in the absence of pre-illumination exhibits a biphasic decay pattern–a faster decay phase (with a half-life, *t*_50%_, of approximately 10 s), which then transitions to a long-lived residual phase which does not decay on the 70 s time window measured. Notably, 30 min of pre-illumination results in almost complete suppression of this slow phase. The very long lifetime (≫ 70 s) of this residual PIA signal suggests it originates from relatively unreactive charges. These unreactive species are observed to have a similar absorption spectrum as reactive species (Figure S8). Figure 2b plots the normalized PIA traces of the fast decay phase (i.e. after subtracting the residual signal at 70 s) for F-BiVO_4_ for different preillumination periods; it is apparent that pre-illumination has no significant impact on the kinetics of this fast decay phase, assigned as is discussed further below, to reactive holes. To minimize the impact of the unreactive charges, and to collect data most relevant to continuous solar irradiation conditions, in all subsequent studies herein, sufficient pre-illumination was employed to suppress the residual PIA signal associated with unreactive charges.

**Figure 3:**
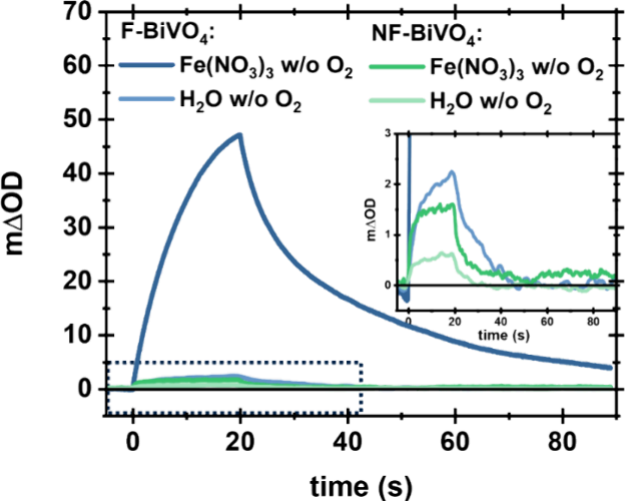
PIA traces in anaerobic 10 mM Fe (NO_3_)_3_ and DI-water probed at 550 nm of F-BiVO_4_ (blue) and NF-BiVO_4_ (green). Anaerobic condition is obtained by purging solution with N_2_ for 30 min to remove O_2_ in the solution. The inset figure represents the zoom of dashed area.

We turn now to PIA studies measured in the presence of 10 mM Fe(NO_3_)_3_ as a reversible electron acceptor (i.e., operando conditions for water oxidation), as shown in Figure 3. Measurements were conducted under anaerobic conditions to avoid possible competing effects resulting from electron transfer to oxygen. All measurements were undertaken following pre-illumination, as discussed above; in the presence of Fe^3+^, a shorter pre-illumination time was required to reach an invariant PIA signal (Figure S9). Compared to the signal in the absence of an electron scavenger (Fe^3+^ or oxygen), the amplitude of PIA signal of F-BiVO_4_, indicative of hole accumulation, increases substantially (up to 25-fold) in the presence of Fe^3+^ (Figure 3). Compared to NF-BiVO_4_, the PIA signal for F-BiVO_4_ is 30-fold larger, yielding a maximal PIA signal amplitude of ∼45 mΔOD and accompanied by an increase in the PIA signal decay half-time from 1.8 to 10.0 s. Employing an effective “roughness factor” for our films of ∼10, determined from our SEM data and particle concentration (see ESI for detailed calculation), we estimate this PIA signal amplitude corresponds to ∼65 holes/nm^2^, a remarkably high surface hole density, as we discuss further below. BET data for particles show a lower hole density estimation of ∼20 holes/nm^2^ (see discussion in the ESI). Nevertheless, these values consistently demonstrate a significant difference in hole density between F-BiVO_4_ and NF-BiVO_4_.

The increase in the yield and lifetime of F-BiVO_4_ holes in the presence of Fe^3+^ clearly indicates suppressed recombination losses, consistent with the electron-accepting function of Fe^3+^. Supporting this conclusion, bubble formation was observed during our PIA studies of F-BiVO_4_ in the presence of Fe^3+^, confirming the operando nature of these experiments. For NF-BiVO_4_, the increase in amplitude and lifetime for the PIA signal with Fe^3+^ addition is less pronounced. Overall, in the presence of Fe^3+^, we observed that faceting results in a 30-fold increase in the density of accumulated BiVO_4_ holes, as well as a 5-fold increase in their decay half times, consistent with the increase in AQE for oxygen evolution shown in Figures 1 and S2.^17^

**Figure 4:**
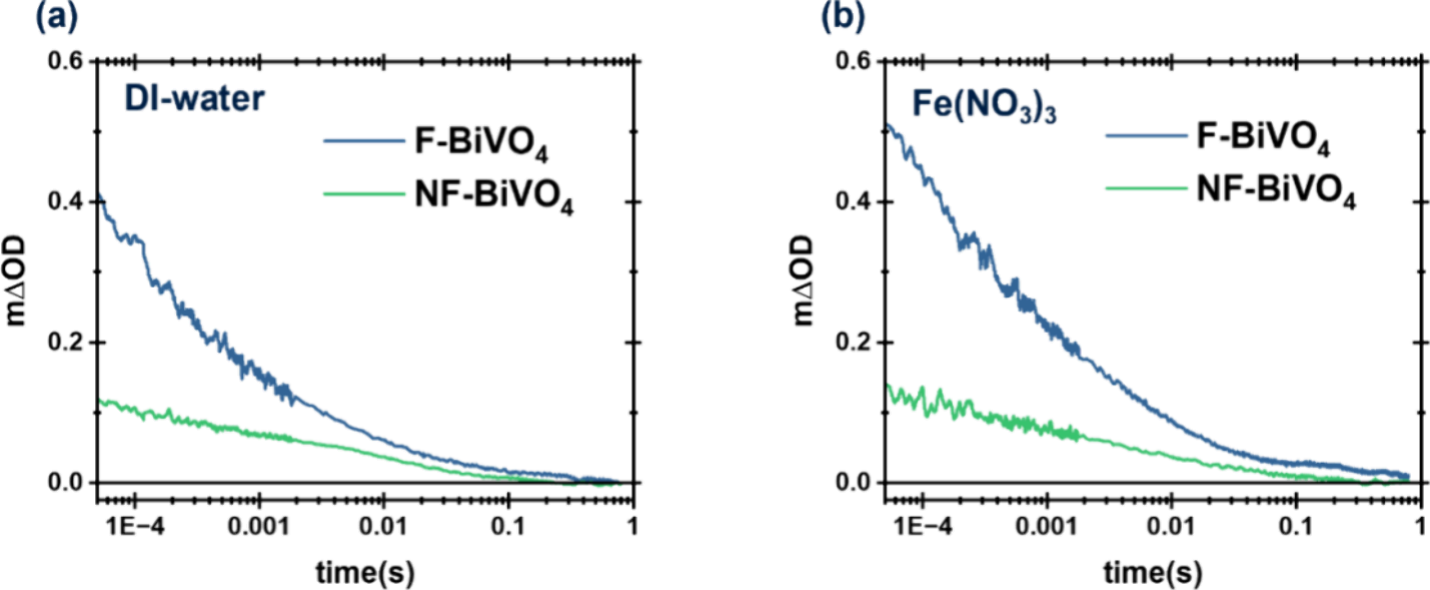
TAS traces probed at 600 nm of F-BiVO_4_ (blue) and NF-BiVO_4_ (green) in (a) DI-water and (b) 10 mM Fe(NO_3_)_3_ solution as electron scavenger. Laser intensity: 554 μJ/cm^2^.

Transient Absorption Spectroscopy (TAS) employing a short (ns) excitation laser pulse (λ = 355 nm) was used to extend our studies to shorter time scales. Typical data for F- and NF-BiVO_4_ in the absence and presence of Fe^3+^ are shown in Figure 4. We note that the relatively intense laser pulse conditions employed in these TAS studies result in accelerated bimolecular recombination losses and, therefore, faster decay kinetics than those we observe in our operando PIA studies. TAS measurements as a function of laser intensity (Figures S10 and S11) indicate a saturation of signal intensity at high laser powers. This observation suggests bimolecular losses on faster time scales than <50 μs. In any case, it is apparent from Figure 4 that F-BiVO_4_ shows higher signal amplitudes at all times measured (i.e., starting from 50 μs) compared to NF-BiVO_4_, indicating that facet-induced charge separation occurs on faster (<50 μs) time scales. The addition of Fe^3+^ results in a small increase in the initial signal amplitude, suggesting fast electron extraction from the BiVO_4_ to Fe^3+^ in <50 μs, although we note that further study directly probing BiVO_4_ electron signals would be needed to substantiate this conclusion.

**Figure 5:**
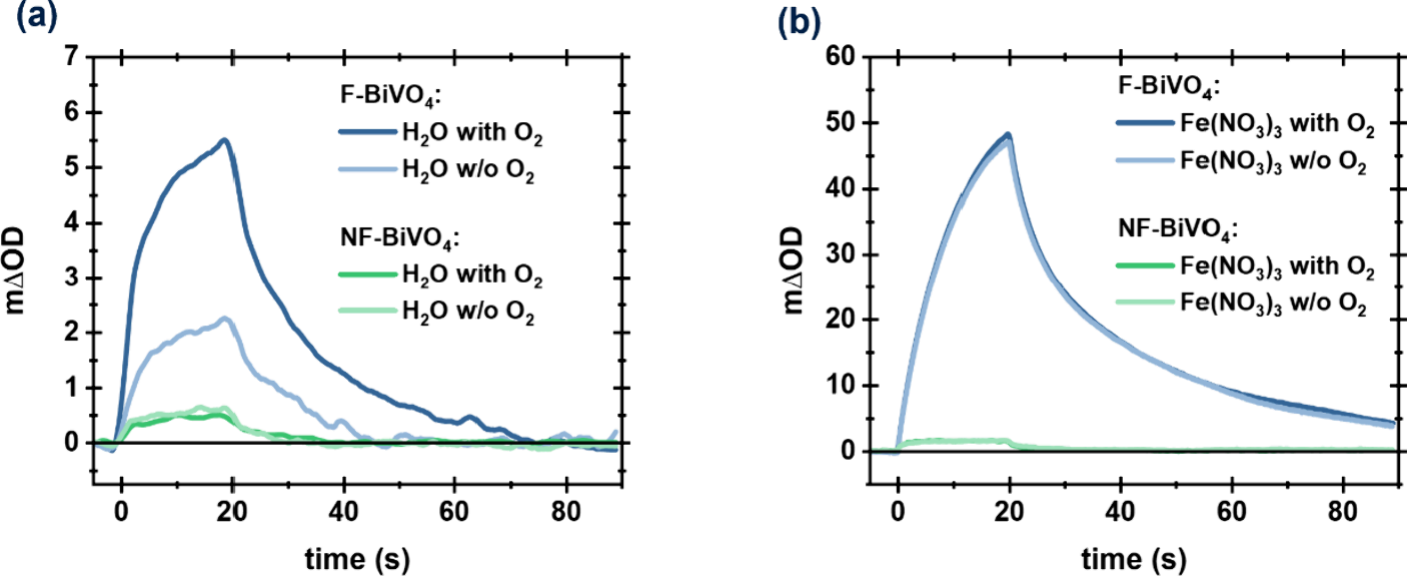
Comparison of PIA traces probed at 550 nm of F-BiVO_4_ (blue) and NF-BiVO_4_ (green) in (a) aerobic and anaerobic DI-water solution, (b) aerobic and anaerobic 10 mM Fe(NO_3_)_3_ solution.

The kinetic competition between Fe^3+^ and the water oxidation product, oxygen, for electron extraction is a significant challenge for improving oxygen evolution performance.^29^ Consequently, we undertook PIA measurements in DI-water and Fe(NO_3_)_3_ solution under aerobic versus anaerobic conditions. As shown in Figure 5, PIA data collected for NF-BiVO_4_ exhibited no discernible difference between aerobic and anaerobic conditions. In contrast, for F-BiVO_4_ in DI-water, both the lifetime and amplitude of the PIA hole signal are increased by the presence of oxygen, suggesting that oxygen can act as an electron acceptor and thus suppress recombination losses (Figure 5a). However, these increases in the presence of oxygen are an order of magnitude smaller than those observed with the addition of Fe(NO_3_)_3_. Consistent with this conclusion, no discernible difference can be observed between aerobic and anaerobic Fe(NO_3_)_3_ solutions for both BiVO_4_ samples (Figure 5b). It can thus be concluded that while oxygen (at atmospheric pressure) can function as an electron acceptor for F-BiVO_4_, the kinetics of O_2_ reduction are too slow to compete significantly with electron transfer to Fe^3+^. This is consistent with the efficient AQE for oxygen evolution observed for our F-BiVO_4_ (Figure 1), as it indicates that oxygen generated by OER reaction will not compete with the electron-accepting function of Fe^3+^.

## Discussion

Facet-engineering has previously been shown to be an effective strategy for enhancing the photocatalytic activity of BiVO_4_ particles for OER in the presence of a reversible electron acceptor, such as Fe^3+^.^17^ In this work, we explored the origin of this enhanced photocatalytic function in terms of its impact on the kinetics of BiVO_4_ valence band holes. Our studies have employed operando PIA spectroscopy, monitoring the accumulation and decay kinetics of BiVO_4_ holes generated by quasi-continuous (20 s duration), one sun equivalent pulsed LED light excitation. Comparing PIA data for F- and NF-BiVO_4_, we quantify the increases in the accumulated density, lifetime and quantum yields of long-lived BiVO_4_ holes induced by faceting, as summarized in Table S1. We also find that the addition of Fe^3+^ results in further increases in all these parameters. Thus, both Fe^3+^ and faceting result in substantial and additive suppression of charge recombination losses in BiVO_4_ photocatalyst particles. These impacts combine to yield the remarkably high (63%) AQE observed for the OER reaction for F-BiVO_4_ in the presence of Fe^3+^.

**Scheme 1:**
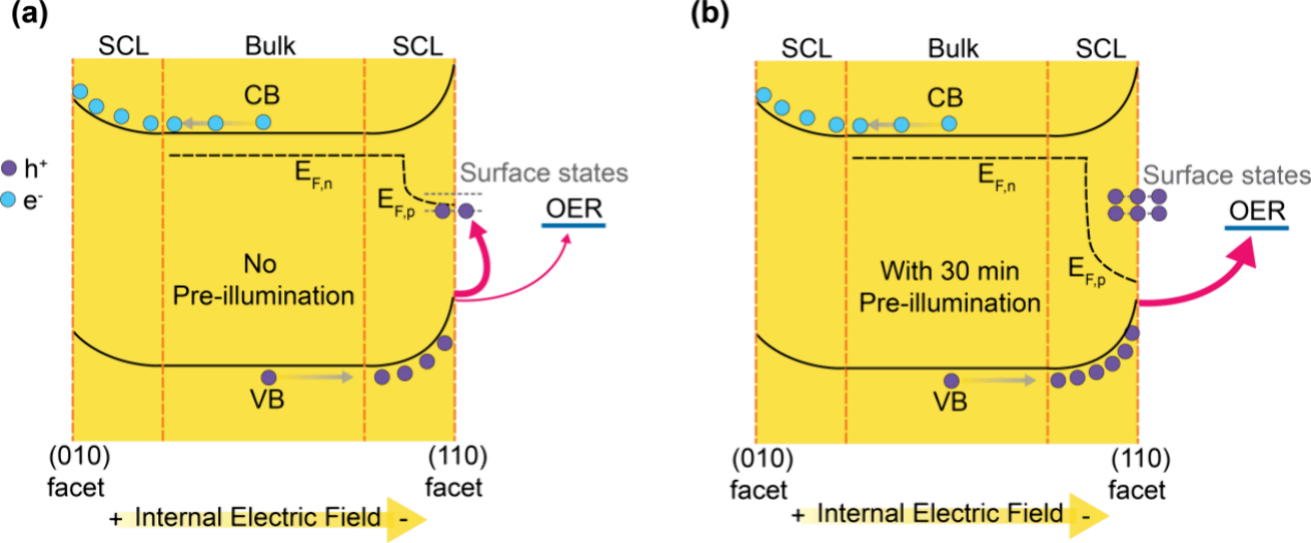
Schematic of the Effect of Pre-Illumination on the Filling of Surface States in F-BiVO_4_ In the absence of pre-illumination (a) a significant fraction of photogenerated holes is trapped in unreactive mid-gap states. (b) Following pre-illumination, these trap states are fully oxidised (filled with holes), preventing further hole trapping and increasing the efficiency of the OER.

Before discussing in more detail the impact of faceting on the hole dynamics in our BiVO_4_ photocatalysts, we discuss our observation that pre-illumination can significantly impact these dynamics. It is important to note that the observation of exceptionally long-lived charges during our initial (without pre-illumination) PIA measurements of F-BiVO_4_ indicated biphasic PIA decay kinetics as illustrated in Figure 2a: a fast (∼10 s) decay phase assigned to reactive holes, and a slow (≫ 70 s) decay phase assigned to unreactive, deeply trapped charges. The amplitude of this slow phase can be fully suppressed by pre-illumination. To explain this phenomenon, we propose a model for the pre-illumination effect on these charges, as illustrated in Scheme 1. Following a sufficient period of pre-illumination (around 30 min), the deep hole traps become fully oxidized (i.e., filled by holes), leading to the accumulation of only reactive holes driving OER.

The function of midgap surface states on metal oxide photoelectrodes and photocatalysts is still controversial. Some studies indicate such states can act as recombination centers that facilitate the recombination of electrons and holes.^30,31^ On the other hand, surface states have also been suggested to be beneficial to catalysis, as the trapping process could compete with recombination and increase the lifetime of charge carriers.^32^ In addition, some electrochemical impedance studies have suggested that water oxidation is driven primarily by such trapped holes rather than by holes directly from the valence band.^33^ In contrast, other studies, including our own, have focused on the dominant role of valence band surface holes in driving water oxidation catalysis, with intragap trap/defect states in the space charge layer primarily being involved in space charge layer formation and reversible electron trapping.^28,30,34^ In BiVO_4_, deep hole traps are predominantly linked to intrinsic defects such as oxygen vacancies, vanadium vacancies, and bismuth interstitials. Notably, surface oxygen vacancies are especially prevalent and play a crucial, yet still not fully understood, role in the material’s photocatalytic performance.^35^

Our results herein indicate that, for the studied F-BiVO_4_, these surface states primarily function as deep hole trap states. The very long lifetime of these trapped holes (≫10 s) indicates they are less reactive than surface valence band holes and also do not function as rapid recombination centers. Pre-illumination results in a filling/passivation of these states, without impacting upon the kinetics of water oxidation (Figure 2b). We note these states are also likely to be present in NF-BiVO_4_; however, the smaller signals (and low yields of accumulated holes) most likely prevent us from resolving these. In any case, these trap states are readily passivated by pre-illumination, such that they do not appear to significantly limit the steady state performance of our F-BiVO_4_ samples, consistent with their high photocatalytic activity.

We now explore the impact of facet-engineering on enhancing charge accumulation and extending the lifetime of reactive species. A significant finding of this study is the 4-fold increase in the charge accumulation amplitude of F-BiVO_4_ compared to NF-BiVO_4_, even in anaerobic conditions and without adding Fe^3+^ acceptor. Additionally, facet-engineering increases the lifetime of these reactive holes 3-fold (in anaerobic water, see Table S1). This phenomenon becomes markedly more pronounced with the introduction of Fe^3+^. The amplitude of accumulated charges in F-BiVO_4_ experiences a substantial increase to approximately ∼45 mΔOD, which is ∼30 times greater than that of NF-BiVO_4_ (∼1.5 mΔOD). Furthermore, the lifetime of these charges also sees a significant extension due to the facet structure (∼5-fold in the presence of Fe^3+^), as shown in Figure 3 and Table S1. We attribute these improvements in hole accumulation and lifetime to enhanced charge separation and suppressed recombination due to hole stabilization on the (110) facet surface.

**Scheme 2:**
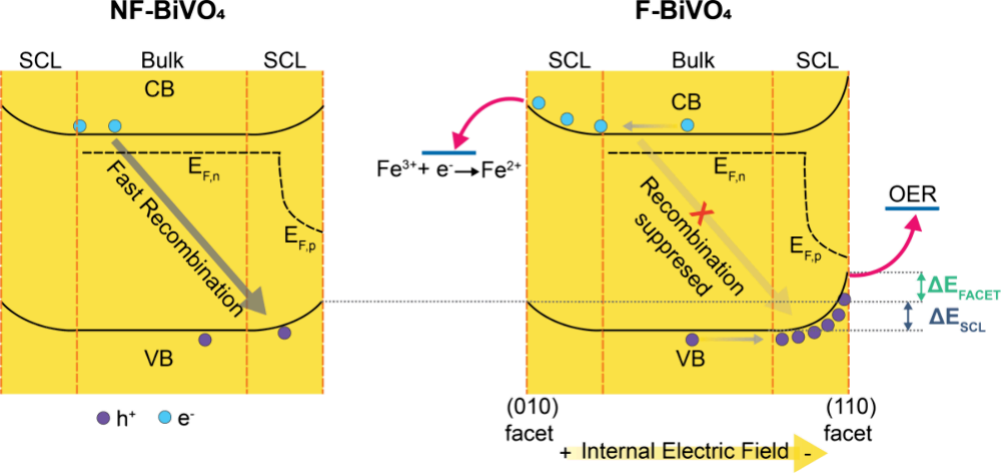
Schematic Illustrating the Effect of Faceting on the Charge Dynamics of BiVO_4_ Photocatalysts Faceting results in an additional energetic stabilisation of holes on the (110) surface, suppressing back electron -hole recombination and increasing the efficiency of OER, charge separation and water oxidation reaction.

As illustrated in Scheme 2, holes on the surface of F-BiVO_4_ are stabilized by both the space charge layer (SCL) formation resulting from Fermi level equilibration with the electrolyte and by the surface dipole resulting from the (110) facet termination. The dominant recombination pathway competing with OER by these surface holes is back electron–hole recombination (BER): the recombination of surface holes with bulk electrons. If, for simplicity, we ignore tunnelling pathways for BER, then BER can be described as resulting from the thermally driven transfer of holes from the surface back into the BiVO_4_, where these holes then undergo rapid recombination with the electron majority carriers in this n-doped material. Given that the recombination time of bulk charge carriers in BiVO_4_ is ca. 1 ns,^28^ and our observed surface hole lifetime is ∼10 s, this requires an 10^10^ equilibrium constant between bulk and surface holes. This lifetime gain thus requires an energetic stabilization of holes on the F-BiVO_4_ surface of ∼2.303*k*_B_*T*log10–590 meV.^4^ We note this energetic offset is a lower limit; empirically we have found that achieving 10 lifetime gain in solar conversion devices typically costs ∼90 meV, or ∼900 meV for 10^10^ gain.^4^ In summary, the remarkably long-lived (10 s) hole we observe accumulating on our F-BiVO_4_ particles implies an energetic stabilization relative to bulk of 600–900 meV.

The majority of this energetic stabilization can be expected to result from space charge layer formation (Δ*E*_SCL_), independent of surface faceting. Comparison between open-circuit potential (OCP) and flat-band potential (*V*_FB_) of BiVO_4_ photoanodes^20,21^ would suggest that the band bending resulting from space charge layer formation due to equilibration with the electrolyte is 400–800 meV. This is consistent with even our NF-BiVO_4_ exhibiting the measurable accumulation of surface holes with ∼1 s lifetimes. The further ca. 10-fold increase in hole accumulation density and lifetime we observe for F-BiVO_4_ is indicative of an additional energetic stabilization of 50–90 meV resulting from the (110) facet termination (Δ*E*_FACET_).^4^ This energetic stabilization is comparable to contact potential differences measured by KPFM for faceted BiVO_4_, which range from 20 to 50 meV.^17,18^ We note such KPFM measurements were not conducted operando, which may explain their rather smaller values. We also note that the length scale of band bending associated with space charge layer formation is likely to be longer than that induced surface facet energies (as illustrated in Scheme 2), which may also affect the impact of their respective band bending in suppressing BER. In any case, we can conclude that the combined effects of space charge layer formation and surface facet energies are sufficient to enable the remarkably high yields of long-lived holes on BiVO_4_.

**Figure 6:**
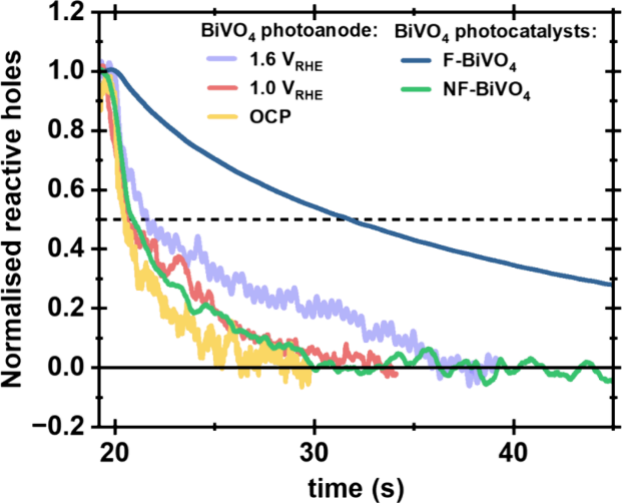
Comparison of PIA decay kinetics probed at 550 nm for a non-faceted BiVO_4_ photoanode fabricated by spin coating in 0.1 M potassium phosphate buffer pH 6.7 under different anodic biases, and BiVO_4_ photocatalysts (NF-BiVO_4_ and F-BiVO_4_) in DI-water with 10 mM Fe (NO_3_)_3_. All data employed ca. 1 sun pulsed irradiation.

We conclude our analysis of lifetime gain and energetic loss in our BiVO_4_ photocatalysts by comparing our measured hole lifetimes with those observed on non-faceted BiVO_4_ photoanodes at open circuit and under applied bias, as illustrated in Figure 6. It is apparent that our NF-BiVO_4_ exhibits similar surface hole decay kinetics to our photoanode at open circuit, which as expected, indicates that non-faceted photoanodes and particles exhibiting similar hole decay kinetics in the absence of applied bias. In both cases these kinetics are primarily dominated by BER. In contrast, it is apparent that our F-BiVO_4_ exhibits hole decay kinetics significantly (∼5 times) slower than the BiVO_4_ photoanode at strong anodic bias; in both these cases the kinetics can be assigned primarily to OER. This indicates that water oxidation proceeds more slowly on F-BiVO_4_ than on our BiVO_4_ photoanode. Consistent with its OER kinetics, F-BiVO_4_ also demonstrates a much higher surface hole density —approximately 65 h^+^/nm^2^ (with a roughness factor of ∼10), compared to only 5 to 6 for the BiVO_4_ photoanode (with a roughness factor of ∼1), as detailed in the ESI.^25^ The slower OER kinetics observed for our F-BiVO_4_ is tentatively assigned to the higher (less oxidizing) surface valence band edge induced by the (110) facet energy. Literature suggests that facet-engineering can shift the valence band (V_B_) position at the interface toward less negative values (vs vacuum), aligning it closer to the water oxidation potential.^15,19^ Consequently, this realignment would reduce the driving force available to holes to drive water oxidation. It may also be associated with the accumulation of some subsurface holes on our F-BiVO_4_, with these subsurface holes being unable to drive OER directly. Additionally, the larger hole accumulation we observe for F-BiVO_4_ may also be due to the use of Fe(NO_3_)_3_ as an electron scavenger to extract electrons from each particle, in contrast to our BiVO_4_ photoanode, where electron extraction requires diffusion to the back contact under the applied anodic potential.

Finally, we note the apparent superior stability of F-BiVO_4_ in comparison to typical BiVO_4_ photoanodes. BiVO_4_ photoanodes often suffer from degradation issues, particularly under harsh conditions. In contrast, the F-BiVO_4_ particles employed herein retained their OER activity, even in highly acidic Fe(NO_3_)_3_ solutions (pH ∼ 2) for extended periods of time (up to 20–30 h).^17^ Similarly, during our PIA measurements, which spanned several hours over multiple days under working conditions, we observed no detectable changes in our PIA data. This increased stability may originate from the higher crystallinity of the nanoparticles relative to typical BVO_4_ photoanodes, as previously reported,^36^ or potentially because the holes in F-BiVO_4_ are less energetic, reducing their driving force not only for the OER but also for self-oxidation of BiVO_4_, given that these two values are closely aligned.^37^

Our studies indicate that while faceting induces a shift in the surface hole energetics, the surface facets themselves appear to remain relatively unchanged during the OER process. This suggests that surface reconstruction is unlikely to play a significant role in the function or stability of the F-BiVO_4_ under the conditions we investigated. In electrocatalysis, surface reconstruction has been shown to play an important role in the performance of several catalyst materials.^38^ Under operando conditions, the surface structure of such active material can undergo significant changes; this transformation can dramatically influence the catalytic activity and stability of the material. For example, copper-based catalysts often transition from Cu(111) to Cu(100) facets due to structural reconstruction driven by reaction intermediates and the surface polarization.^39,40^ Similarly, electrocatalysts based on transition metal oxides are sometimes considered precatalysts, as the active form is obtained only after postsurface reconstruction.^38^ Interestingly, it has also been shown that in some cases distinct facets experience different types of surface reconstructions.^41^ In BiVO_4_, differences in thermodynamic stabilities may arise due to the different band alignments associated with various facets.^42^ Further, alterations to the surface termination or composition of a single facet have been shown to significantly impact band alignment, without necessitating changes to the atomic planes exposed on the surface.^21^ Despite the prevalence of such transformations in many systems, in the case of F-BiVO_4_, while different facets may exhibit varied thermodynamic stabilities due to changes in band alignments, our observations imply that alterations to the exposed surface are not significant during OER, at least over the time scales (hours to days) studied herein.

## Conclusions

In this study, we employ PIA and TAS to explore the impact of facet-engineering on the charge carrier dynamics in BiVO_4_ photocatalysts for water oxidation in the presence of the electron acceptor Fe(NO_3_)_3_. Our operando PIA spectroscopic analyses indicate that facet-engineering of BiVO_4_ photocatalyst particles can result in a 30-fold enhancement in the density of accumulated long-lived, reactive holes and a 5-fold increase in their lifetime. These findings align well with the 70-fold enhancement observed in OER performance with faceting. These results clearly demonstrate the ability of facet engineering to increase both the yield and lifetime of the long-lived holes required to drive water oxidation.

Our PIA studies further indicate a clear dependence of long-lived hole kinetics upon pre-illumination. This is attributed to the presence of deep hole traps, which become filled, and thus passivated, by prolonged irradiation. PIA studies in the presence/absence of oxygen indicate that while oxygen can also accept photogenerated electrons from the BiVO_4_ photocatalysts, the kinetics of this oxygen reduction are much slower than electron transfer to Fe(NO_3_)_3_, such that this is not a significant competing pathway (at least the 10 mM Fe(NO_3_)_3_ concentration employed herein).

The long-lived holes generated on faceted BiVO_4_ drive water oxidation on the 10 s timescale. This hole lifetime is estimated to be 10^10^ longer than the lifetime of bulk photogenerated holes in this n-type semiconductor (where holes are minority carriers). This lifetime gain is indicative of a hundreds of meV of band bending at the semiconductor surface–resulting in part from Fermi level equilibration between the semiconductor and electrolyte, and in part from additional band bending induced by facet engineering due to surface dipole effect. This energetic loss (i.e.: stabilisation) enables the accumulation of surface holes with lifetimes long enough to drive efficient water oxidation. This ability to drive water oxidation despite this large energetic loss is attributed to the deep valence band of BiVO_4_, such that even after this energetic loss, surface holes retain a significant overpotential for the OER reaction. Appreciation of this lifetime gain requirement, and the energetic loss inherent in gaining lifetime, is likely to be critical to further applications of facet engineering to enhance the performance of photocatalysts for solar-driven fuel synthesis.^6^

## Supporting Information Available

Additional information including BiVO_4_ preparations, experiment procedures (SEM, XRD, UV–vis, AQE, KPFM, operando PIA and TAS), and detailed explanation mentioned in the main text (PDF)
