# Supplementary material for: Facet-Engineered BiVO4 Photocatalysts for Water Oxidation: Lifetime Gain Versus Energetic Loss
Source: J Am Chem Soc. 2024 Sep 21;146(39):27080–9. doi: 10.1021/jacs.4c09219 (PMC11450740; doi:10.1021/jacs.4c09219)
Supplement: Supplementary file 1 — ja4c09219_si_001.pdf [file ja4c09219_si_001.pdf]

Facet-Engineered $BiVO_4$ Photocatalysts for Water Oxidation: Lifetime Gain versus Energetic Loss

Tianhao He[1], Yue Zhao[2], Daniele Benetti[1,*], Benjamin Moss[1], Lei Tian[1], Shababa Selim[1], Rengui Li[2], Fengtao Fan[2], Qian Li[2] Xiuli Wang[2,*], Can Li[2], James R. Durrant[1,*]

Table of Content

Experimental Section:..2

Figure S1. SEM images of F-$BiVO_4$ films with scale bar of (a) 500 nm, (b) 1 μm and (c) 2 μm. SEM images of NF-$BiVO_4$ films with scale bar of (d) 500 nm, (e) 1 μm and (f) 2 μm. 6

Figure S3. Morphology images in KPFM of (a) F-$BiVO_4$ and (c) NF-$BiVO_4$. 3D-CPD images in KPFM of (b) F-$BiVO_4$ and (d) NF-$BiVO_4$. (e) CPD value of F-$BiVO_4$ and NF-$BiVO_4$ for normalised cross section. (f) Average value of CPD on different facets of F-$BiVO_4$ and surface of NF-$BiVO_4$. 7

Figure S2. Apparent Quantum Efficiency (AQE) for water oxidation of F-$BiVO_4$ and NF-$BiVO_4$ photocatalyst dispersion in 10 mM $Fe(NO_3)_3$ solution (used as electron acceptor) under 300 W Xe light source with 420 nm band-pass filter. 7

Figure S4. UV-Vis diffuse reflectance spectra of F-$BiVO_4$ (band gap ~ 2.313 eV) and NF-$BiVO_4$ (band gap ~ 2.254 eV) films in air condition...8

Figure S5. X-ray diffraction (XRD) spectra of F-$BiVO_4$ and NF-$BiVO_4$ films. 8

Figure S6. PIA absorption spectra in 10 mM $Fe(NO_3)_3$ as electron scavenger, 20% methanol (MeOH) as hole scavenger and DI-water measured after 20 s irradiation of a F-$BiVO_4$ film with a 365 nm, 1 sun equivalent LED excitation. 9

Figure S7. PIA absorption spectra in 10 mM $Fe(NO_3)_3$ as electron scavenger, 20% methanol (MeOH) as hole scavenger and DI-water measured after 20 s irradiation of a NF-$BiVO_4$ film with a 365 nm, 1 sun equivalent LED excitation. 9

Figure S9. PIA traces in 10 mM $Fe(NO_3)_3$ as electron scavenger probed at 550 nm of F-$BiVO_4$ with 0 min, 1 min and 30 mins pre-illumination. 10

Figure S8. PIA absorption spectrum of F-$BiVO_4$ in DI-water, red points represent maximum amplitude of PIA (~20 s), blue points represent the amplitude of end of PIA measurements (~90 s), yellow points represent the different of amplitude between red and blue data points. 10

Figure S11. TAS traces of NF-$BiVO_4$ in (a) 10 mM $Fe(NO_3)_3$ and (c) DI-water under different laser intensities. TAS amplitude of the optical signal at 50 μs, 100 μs, and 1 ms for NF-$BiVO_4$ in (b) 10 mM $Fe(NO_3)_3$ and (d) DI water, measured under varying laser intensities. 11

Figure S10. TAS traces of F-$BiVO_4$ in (a) 10 mM $Fe(NO_3)_3$ and (c) DI-water under different laser intensities. TAS amplitude of the optical signal at 50 μs, 100 μs, and 1 ms for F-$BiVO_4$ in (b) 10 mM $Fe(NO_3)_3$ and (d) DI water, measured under varying laser intensities. 11

Table S1. Increases in the lifetime, accumulated density, and quantum yields measurements for NF-$BiVO_4$ and F-$BiVO_4$ in different environments...12

References...13

Experimental Section:

Preparation of F-$BiVO_4$ and NF-$BiVO_4$ powders (used for performance measurements):

F-$BiVO_4$: 10.0 mmol of $Bi(NO_3)_3 \cdot 5H_2O$ and 10.0 mmol of NH_3VO_3 were dissolved in 60 mL of 2.0 M nitric acid solution and stirred until complete dissolution. The pH value of the solution was then adjusted to 0.40 with an ammonia solution (25-28 wt.%) under vigorous stirring. Following this, a light yellow $BiVO_4$ slurry was obtained after stirring for 2 hours at room temperature. Subsequently, the mixture was transferred to Teflon-lined stainless-steel autoclaves with a capacity of 100 mL and hydrothermally treated at 473 K for 12 h in the oven. Upon cooling the autoclaves to room temperature, a yellow precipitate was formed. The obtained precipitate was separated by centrifugation, washed with ultrapure water, and then dried at 353K overnight.
NF-$BiVO_4$: In a typical synthetic procedure, 2.0 mmol of $Bi(NO_3)_3 \cdot 5H_2O$ and 1.0 mmol of V_2O_5 were mixed and grinded in an agate mortar. Then the mixture was calcinated at 823 K in the muffle furnace for 10 hours. After cooling to room temperature, the powder was collected.

Preparation of F-$BiVO_4$ and NF-$BiVO_4$ films (used for PIA measurements):

50 mg $BiVO_4$ was dispersed in a mixture of 2 mL isopropanol and 2 mL ethanol, followed by the addition of 1 mL ethanol solution of naphthol (0.25 wt%). The resulting suspension was subjected to ultrasonic processing before being spray-coated onto PMMA (polymethyl methacrylate) substrates (2×1 cm^{-2}) heated to 80 ^oC on a hot plate. Subsequently, the obtained $BiVO_4$/PMMA panels were dried in an oven at 80 ^oC for 8 hours to obtain the final product.

Preparation of $BiVO_4$ photoanode films (used for PIA measurements):

The $BiVO_4$ photoanode films were prepared by spin-coating method, which has been reported previously.[1-3] In the preparation of the $BiVO_4$ solution for spin coating, 0.1455 g (0.30 mmol) of bismuth nitrate pentahydrate was dissolved in 1.50 mL of acetic acid. Subsequently, 0.0768 g (0.29 mmol) of vanadyl acetylacetonate powder was mixed into 5.00 mL of acetylacetone. This mixture was then combined with the bismuth nitrate solution, followed by a rinse with an additional 5.00 mL of acetylacetone. The combined solutions were stirred at ambient temperature for 25 minutes, forming a turquoise sol-gel. Fluorine-doped tin oxide (FTO) glass squares (1.0 × 2.0 cm) underwent a cleaning process involving sonication for 15 minutes in a sequential wash of detergent solution, de-ionized water, acetone, and isopropanol. After air drying, the glass was subjected to ozone treatment for 10 minutes. The construction of the $BiVO_4$ photoanode film involved depositing 14 layers on the FTO glass. The base layer required 0.25 mL of sol-gel to be spin-coated at 2000 rpm for 20 seconds, followed by a calcination step at 500 ℃ for 10 minutes and a subsequent room temperature cooldown for 10 minutes. The following 13 layers were applied at a reduced spin speed of 1000 rpm for 20 seconds per layer. Each layer was calcined and cooled in the same manner, except for the last layer, which was exposed to prolonged calcination at 500 ℃ for five hours before being allowed to cool to room temperature.

Characterization of the samples:

NF-$BiVO_4$ and F-$BiVO_4$ were characterized using X-ray power diffraction (XRD) on a Rigaku D/Max-2500/PC powder diffractometer with Cu-Kα radiation at 40 kV and 200 mA. The XRD patterns were recorded in the range of 10-60° with a step size of 0.02° and a scan rate of 5°/min. UV-visible (UV-vis) diffuse reflectance spectra were obtained using a UV-vis spectrophotometer (JASCO V-650) equipped with an integrating sphere. The morphologies and particle sizes were analysed through scanning electron microscopy (SEM, Quanta 200 FEG, FEI).

Apparent quantum efficiency and oxygen evolution measurements:

The AQE was measured using a closed gas circulation and evacuation system. 200 mg photocatalyst was dispersed in 4 mM $Fe(NO_3)_3$ solution in a Pyrex reaction cell and thoroughly degassed by evacuation in order to drive off the air inside. Then the reaction cell was irradiated by a 300-W Xe lamp (Ushio-CERMAX LX300) with a 420 nm band-pass filter. The amount of evolved O_2 was determined by an on-line gas chromatograph (Shimadzu GC-8A, TCD, Ar carrier).
The incident photons reaching the solution were measured with a calibrated Si photodiode (LS-100, EKO Instruments Co., LTD.). The AQE (φ) was then calculated using the provided equation.:
$\varphi(\%)=(AR/I)\text{x}100$
Where A represents a coefficient (4 for O_2 evolution), R represents the evolution rate of O_2, and I represents the number of photons reaching the reaction solution.

Photo-Induced Absorption (PIA) Spectroscopy:

The PIA setup is similar to the probe-pump setup previously reported[2, 4-5]. The measurements were conducted over a total duration of 90 seconds. The PIA measurements were carried out simultaneously for a duration of 90 seconds, utilizing pulses from a UV LED light (365 nm) with a cycle of 20 seconds on and 70 seconds off. A single shot measurement is used in order to avoid issues related to equilibrium not being reached before subsequent measurements. This setup utilized a Bentham IL1 tungsten lamp as probe, with a specific wavelength of 550 nm chosen through a monochromator. A long pass filter of 530 nm from Comar Instruments was employed to minimise the LED pump light reaching the detector and avoid photoexcitation of the sample. Photon transmission was captured by a Hamamatsu S3071 Si photodiode. The detected signal was logged using a National Instruments (NI USB-6211) DAQ card. A MOSFET (STF8NM50N from STMicroelectronics) and a frequency generator (TG300 from Thurlby Thandar Instruments) controlled the LED pulse. The LED power is controlled by a QL564P power supply from TTi company. The program software is operated through the LabVIEW platform, and further data analysis is processed by Origin Lab software. The absorption of continuous probe light (550 nm) by electronic charges activated by a 365 nm LED pulse results in a reduced transmittance through the sample, manifesting as an increase in absorbance. The change in absorbance is caused by the accumulation of photoinduced charge carriers generated by the band-gap excitation of LED.

Electrolyte solutions:

A 10 mM solution of $Fe(NO_3)_3$ was prepared by dissolving 0.202 g of Iron(III) nitrate nonahydrate (Sigma-Aldrich) in 50 mL of 15 MΩ·cm deionized water, followed by stirring for 5 minutes. Before the measurements were conducted, the oxygen in the electrolyte solutions (DI water and Fe^{3+} electrolyte) was removed by purging it with nitrogen for 30 minutes.

One sun irradiation of 365 nm LED: The equivalent solar irradiance of a 365 nm LED was fixed at approximately 12.6 mW/cm^2. This estimation is derived from our prior research on $BiVO_4$ photoanodes.[2] In these studies, we calibrated the photocurrent produced by BiVO4 under 365 nm LED illumination to match the photocurrent from the photoanode when exposed to AM 1.5 (100 mW/cm^2) sunlight. We consider the adjusted LED power as equivalent to one sun's irradiance for $BiVO_4$ materials.

Transient Absorption Spectroscopy (TAS): TAS measurements were conducted by using a Nd:YAG laser (Big Sky Laser Technologies) as excitation. The excitation pulse (laser power ranging from 28 to 554 μJ/cm^2) was delivered by the laser's third harmonic, producing a 355 nm wavelength at a 0.33 Hz repetition rate. A 0.5 cm diameter liquid light guide directed the laser pulse to the sample. The same probe light setup used for PIA, consisting of the same tungsten lamp and monochromator used for PIA, was utilized. Additionally, a 530 nm long pass filter was employed to minimize the amount of laser pump light and probe light (λ<530 nm) reaching the detector. The signal was amplified (Costronics amplifier box) before being logged by a Tektronics TDS 2012c oscilloscope (for microsecond to millisecond range) and a National Instruments (NI USB-6211) DAQ card (for millisecond to second range). Program of TAS is also operated through LabVIEW software, data analysis is processed in MATLAB software.

Kelvin Probe Force Microscopy (KPFM)

Kelvin probe force microscopy is an advanced technique based on Atomic Force Microscopy (AFM). It measures the local variations of the surface potential by detecting the contact potential differences (CPD) between a sample and a conductive AFM probe (also called the Kelvin tip). The CPD between the tip and the sample is defined as:

$$CPD = \frac{\phi_t - \phi_s}{e}$$

Where Φt and Φs are the work functions of the tip and the sample surface. The precisely determination of CPD is based on a kelvin probe technique. When the tip of the probe is brought close to the surface, prior to contact, the tip and the sample are electrically neutral and share the same local vacuum level. Upon short-circuiting, the Fermi levels are aligned through charge flow and the system reaches an equilibrium state. The charge flow leads to the charging of the tip and sample surfaces, and an electrical force forms in the gap between the tip and sample surface. Meanwhile, the local vacuum levels differ and the corresponding potential difference is known as the CPD. If an applied external bias (V_{DC}) has the same magnitude as the CPD with opposite direction, the surface charges and the electrical force would be nullified. Thus, the CPD is determined by tuning the external bias V_{DC} to nullify the electrical force. By introducing an AC voltage, the nullification can be easily achieved. The amplitude-modulated mode with a Bruker SCM-PIT-V2 probe was adopted during the measurements under ambient conditions. The radius of

the tip was 25 nm. The resonance frequency of the probe is within the range of 50-100 kHz and the mean spring constant is $3Nm^{-1}$. The probe moved under the control of the AFM and produced the CPD image (denoted as the surface potential image). The lift height was adjusted to 50 nm to obtain the best signal-to-noise ratio and minimize the possible cross-talk effect during the KPFM imaging measurement.

BET (Brunauer-Emmett-Teller) surface area analysis

Nitrogen adsorption-desorption measurements were performed at 77.35 K using a Micromeritics ASAP 2020 analyzer, following the degassing of samples at 363 K for 2 hours.

Calculation of surface hole density:

The extinction coefficient of surface holes in $BiVO_4$ is around 420 M^{-1} cm^{-1} according to the studies in our group previously.[2] The roughness factor of the F-$BiVO_4$ sample is around 10, while for NF-$BIVO_4$ is ~6, based on the average size and surface area of particles, according to the literature.[6] Thus, for F-$BiVO_4$, 1 mΔOD optical signal can be converted to 1.43 surface hole density (nm^{-2}), while for NF-$BiVO_4$ 1 mΔOD can be converted to ~2.38 hole density (nm^{-2}).

Details of conversion for F-$BiVO_4$ (Based on Beer-Lambert law):

$$\Delta\ O.D = \varepsilon * \ p_s * (roughness\ factor)$$

$$1 * \Delta\ O.D = \ 420\ M^{-1}\ cm^{-1} * \ p_s * 10$$

$$1 * \Delta\ O.D = \ 4.2 * 10^{19}\ nm^2\ mol^{-1} * \ p_s * 10$$

$$p_s = \frac{1 * \Delta\ O.D * (Avogadro\ constant)}{4.2 * 10^{19} * 10} nm^{-2}$$

$$p_s = \frac{1 * \Delta\ O.D * (6.02 * 10^{23})}{4.2 * 10^{19} * 10} nm^{-2}$$

$$p_s = \ 1.43 * 10^3 * \Delta\ O.D\ nm^{-2}$$

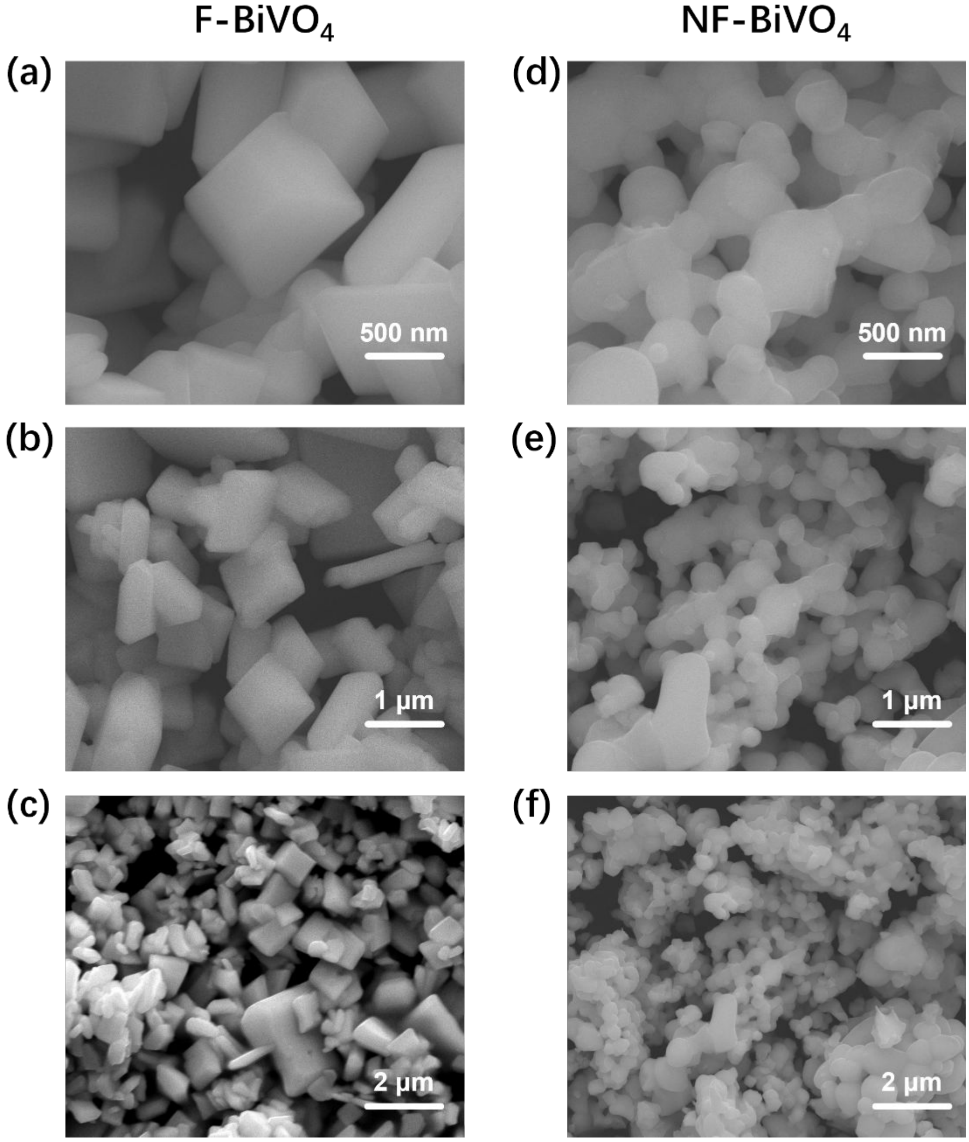

Figure S1. SEM images of F-BiVO4 films with scale bar of (a) 500 nm, (b) 1 μm and (c) 2 μm. SEM images of NF-BiVO$_4$ films with scale bar of (d) 500 nm, (e) 1 μm and (f) 2 μm.

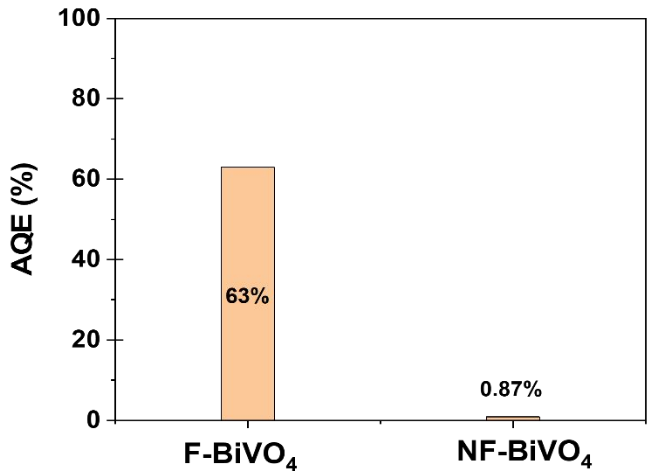

Figure S2. Apparent Quantum Efficiency (AQE) for water oxidation of F-$BiVO_4$ and NF-$BiVO_4$ photocatalyst dispersion in 10 mM $Fe(NO_3)_3$ solution (used as electron acceptor) under 300 W Xe light source with 420 nm band-pass filter.

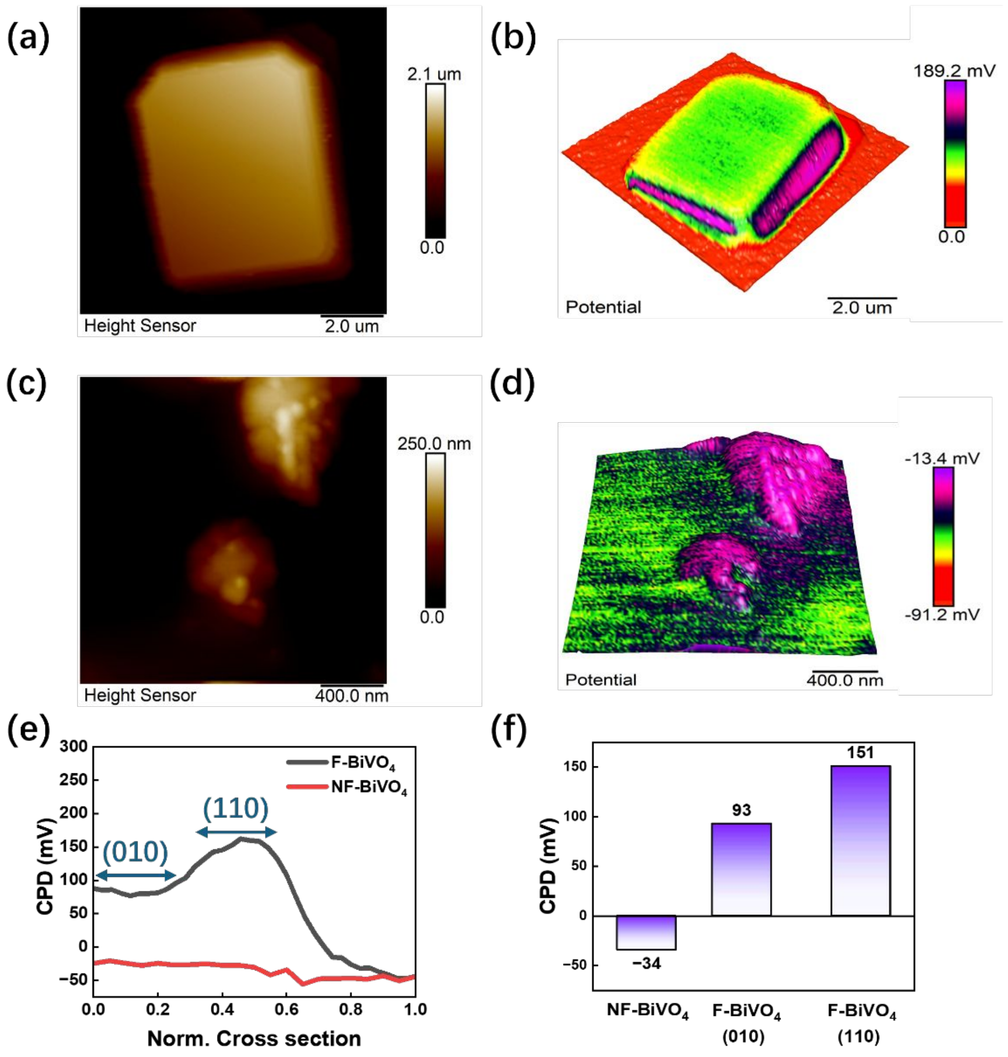

Figure S3. Morphology images in KPFM of (a) F-$BiVO_4$ and (c) NF-$BiVO_4$. 3D-CPD images in KPFM of (b) F-$BiVO_4$ and (d) NF-$BiVO_4$. (e) CPD value of F-$BiVO_4$ and NF-$BiVO_4$ for normalised cross section. (f) Average value of CPD on different facets of F-$BiVO_4$ and surface of NF-$BiVO_4$.

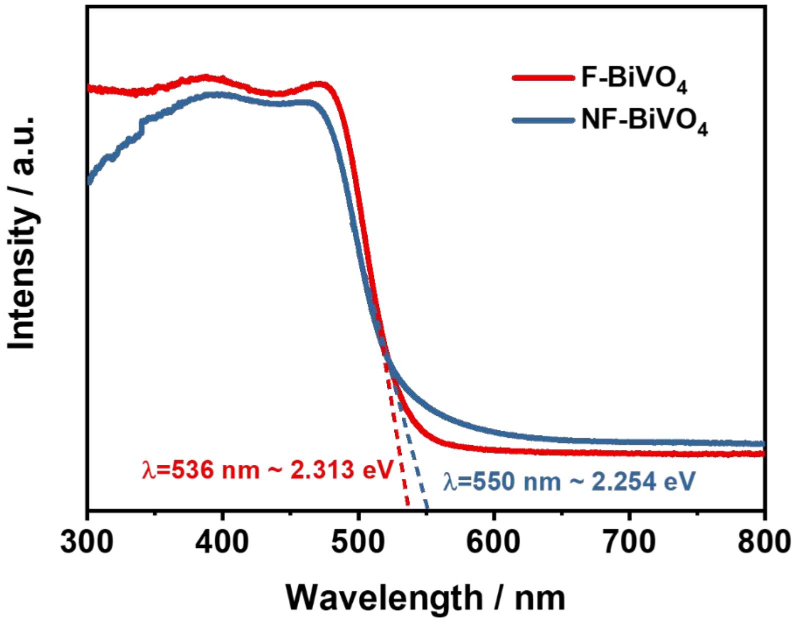

Figure S4. UV-Vis diffuse reflectance spectra of F-$BiVO_4$ (band gap ~ 2.313 eV) and NF-$BiVO_4$ (band gap ~ 2.254 eV) films in air condition.

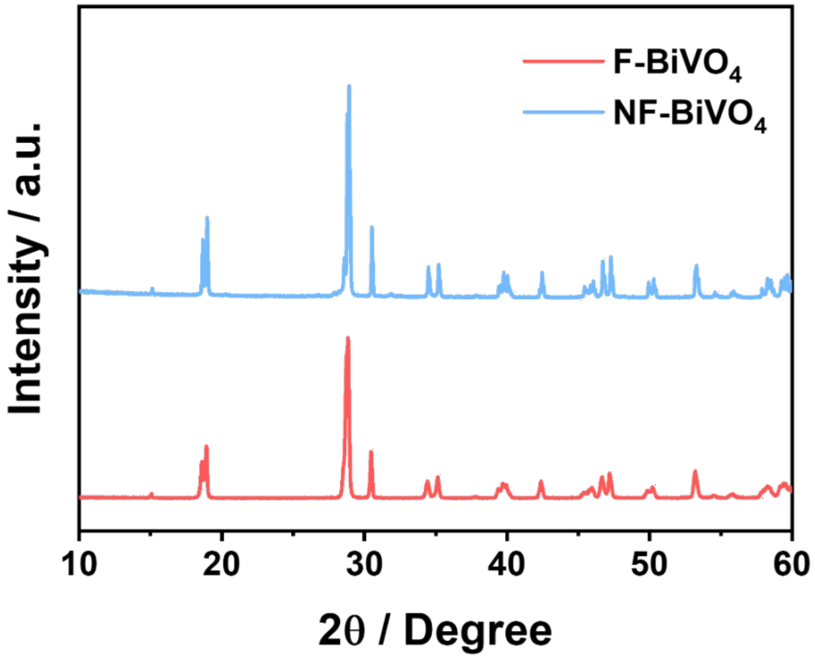

Figure S5. X-ray diffraction (XRD) spectra of F-$BiVO_4$ and NF-$BiVO_4$ films.

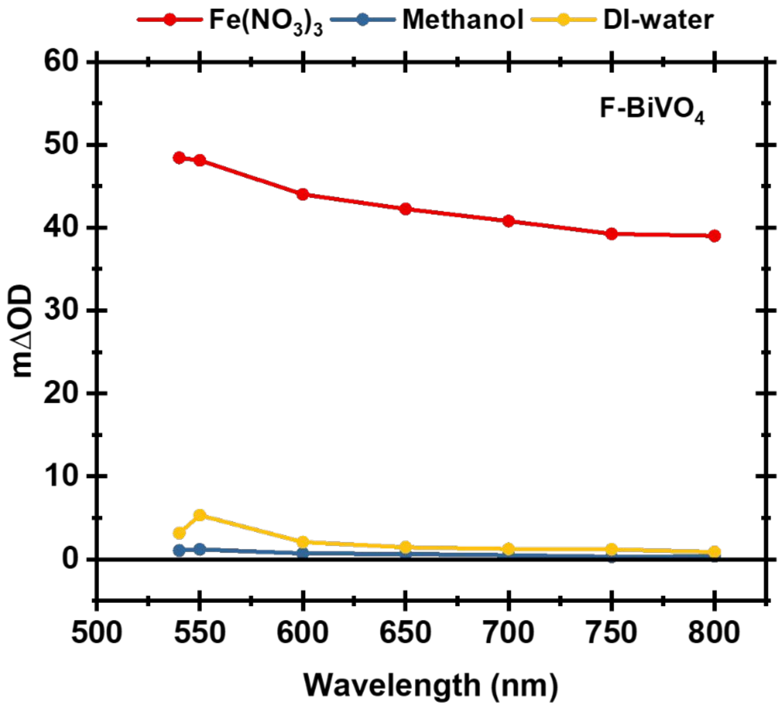

Figure S6. PIA absorption spectra in 10 mM $Fe(NO_3)_3$ as electron scavenger, 20% methanol (MeOH) as hole scavenger and DI-water measured after 20 s irradiation of a F-$BiVO_4$ film with a 365 nm, 1 sun equivalent LED excitation.

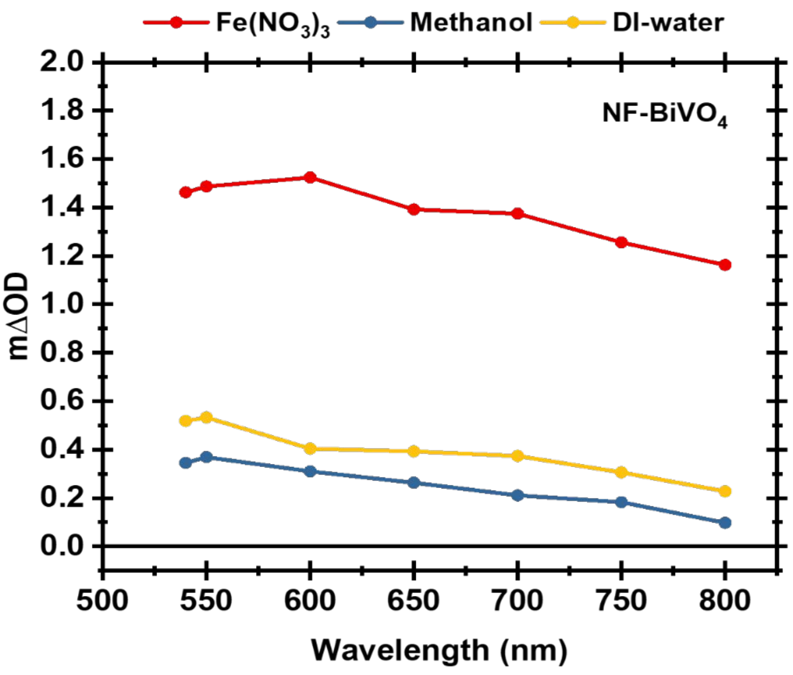

Figure S7. PIA absorption spectra in 10 mM $Fe(NO_3)_3$ as electron scavenger, 20% methanol (MeOH) as hole scavenger and DI-water measured after 20 s irradiation of a NF-$BiVO_4$ film with a 365 nm, 1 sun equivalent LED excitation.

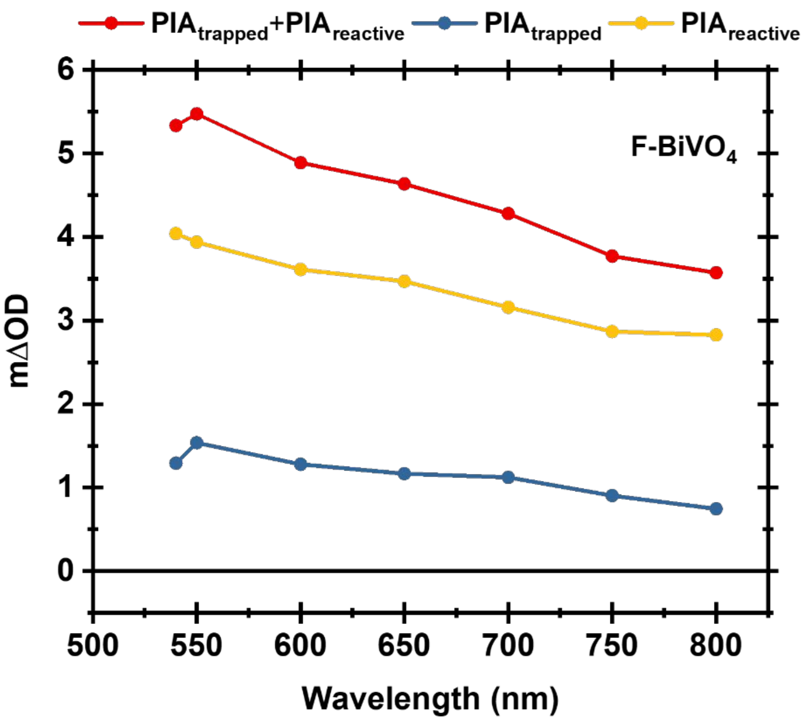

Figure S8. PIA absorption spectrum of F-$BiVO_4$ in DI-water, red points represent maximum amplitude of PIA (~20 s), blue points represent the amplitude of end of PIA measurements (~90 s), yellow points represent the different of amplitude between red and blue data points.

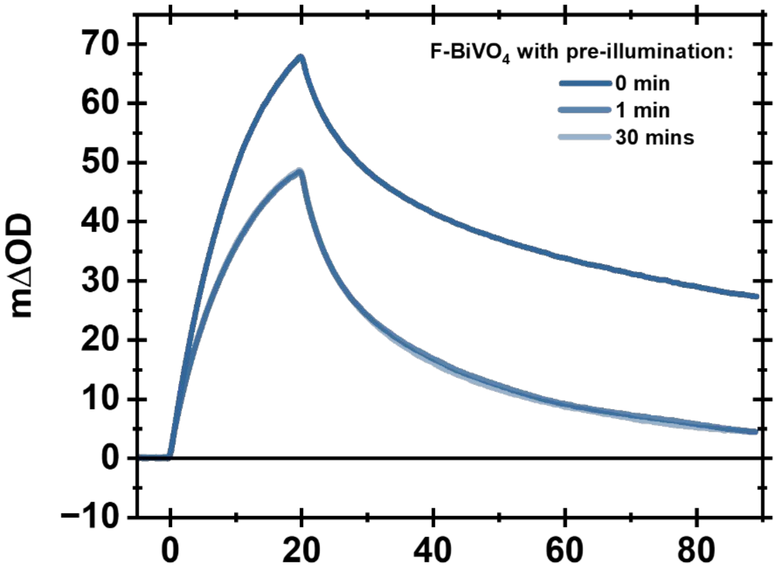

Figure S9. PIA traces in 10 mM $Fe(NO_3)_3$ as electron scavenger probed at 550 nm of F-$BiVO_4$ with 0 min, 1 min and 30 mins pre-illumination.

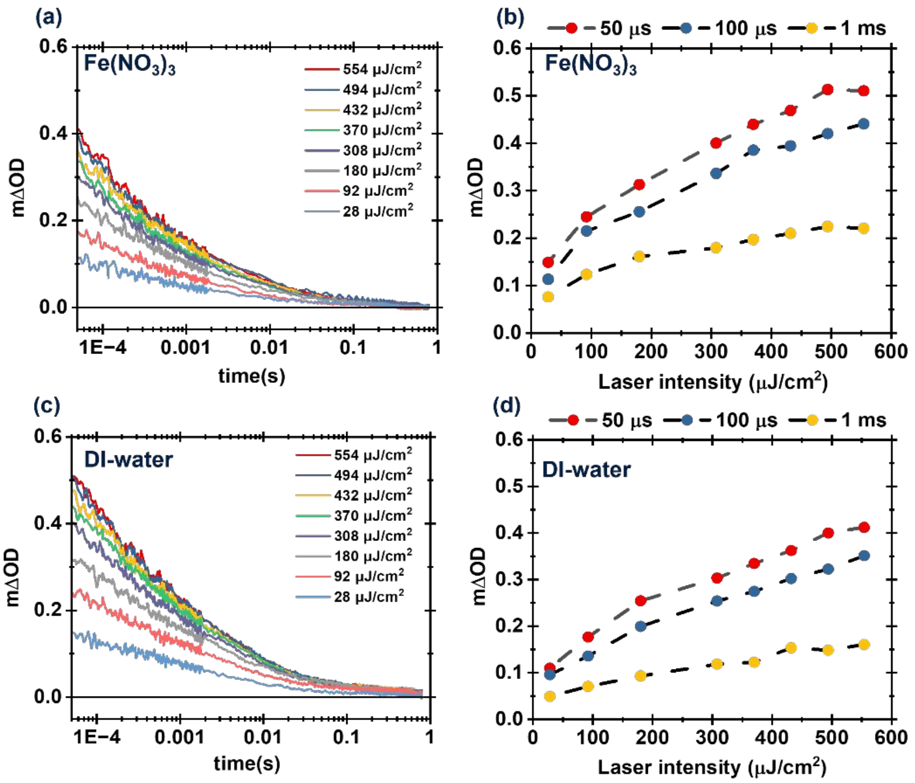

Figure S10. TAS traces of F-BiVO$_4$ in (a) 10 mM Fe(NO$_3$)$_3$ and (c) DI-water under different laser intensities. TAS amplitude of the optical signal at 50 μs, 100 μs, and 1 ms for F-BiVO$_4$ in (b) 10 mM Fe(NO$_3$)$_3$ and (d) DI water, measured under varying laser intensities.

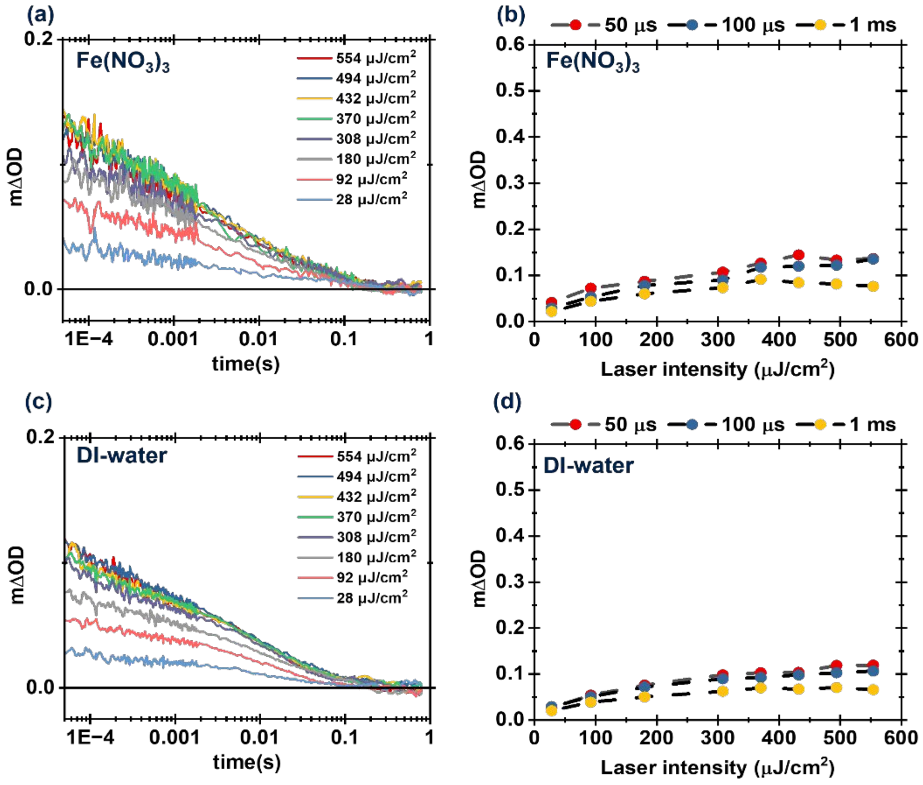

Figure S11. TAS traces of NF-BiVO$_4$ in (a) 10 mM Fe(NO$_3$)$_3$ and (c) DI-water under different laser intensities. TAS amplitude of the optical signal at 50 μs, 100 μs, and 1 ms for NF-BiVO$_4$ in (b) 10 mM Fe(NO$_3$)$_3$ and (d) DI water, measured under varying laser intensities.

	$\tau_{50\%}$ NF-$BiVO_4$ (s)	$\tau_{50\%}$ F-$BiVO_4$ (s)	Max PIA NF-$BiVO_4$ (mΔOD)	Max PIA F-$BiVO_4$ (mΔOD)	h^+ density NF-$BIVO_4$ (h^+/nm^2)	h^+ density F-$BIVO_4$ (h^+/nm^2)	AQE NF-$BiVO_4$ (%)	AQE F-$BiVO_4$ (%)
**Aerobic water**	1.5	7.7	0.52	5.46	1.24	7.81	N/A	N/A
**Anaerobic water**	1.7	5.1	0.62	2.24	1.48	3.20	N/A	N/A
**Aerobic $Fe(NO_3)_3$**	1.8	10.0	1.59	48.08	3.79	68.75	0.62	38.80
**Anaerobic $Fe(NO_3)_3$**	1.8	10.0	1.50	45.00	3.57	64.35	N/A	N/A

Table S1. Increases in the lifetime, accumulated density, and quantum yields measurements for NF-$BiVO_4$ and F-$BiVO_4$ in different environments.

	F BET (m_2/g)-BVO	h^+ density (h^+/nm^2) from BET
**F-$BiVO_4$**	1.064	19.35
**NF-$BiVO_4$**	1.181	0.5

Table S2. BET surface area data and corresponding hole density values.

To calculate the roughness factor (R) based on the BET measurements:

$$R = \frac{BET\ surface\ area\ * mass}{geometric\ area}$$

Based on the BET measurements and considering a mass of 6.25 mg, the roughness factors for F-$BiVO_4$ and NF-$BiVO_4$ are 33 and 40, respectively. Thus, the calculated hole density values from BET data are approximately 19 h/nm² for F-$BiVO_4$ and 0.5 h/nm² for NF-$BiVO_4$.

The roughness factor values estimated by BET are higher than those estimated by SEM analysis which is expected since films typically exhibit lower roughness than particulate powders. While determining the exact number of holes per nm² is challenging and not the primary focus of this work, it is clear that there is a significant difference in hole accumulation between the faceted and non-faceted samples.

References

1. Ma, Y.; Pendlebury, S. R.; Reynal, A.; Le Formal, F.; Durrant, J. R., Dynamics of photogenerated holes in undoped BiVO4 photoanodes for solar water oxidation. *Chem. Sci.* **2014,** *5* (8), 2964-2973.
2. Ma, Y.; Mesa, C. A.; Pastor, E.; Kafizas, A.; Francas, L.; Le Formal, F.; Pendlebury, S. R.; Durrant, J. R., Rate law analysis of water oxidation and hole scavenging on a BiVO4 photoanode. *ACS Energy Letters* **2016,** *1* (3), 618-623.
3. Li, B.; Oldham, L. I.; Tian, L.; Zhou, G.; Selim, S.; Steier, L.; Durrant, J. R., Electrochemical versus Photoelectrochemical Water Oxidation Kinetics on Bismuth Vanadate (Photo) anodes. *Journal of the American Chemical Society* **2024,** *146* (18), 12324-12328.
4. Le Formal, F.; Pastor, E.; Tilley, S. D.; Mesa, C. A.; Pendlebury, S. R.; Grätzel, M.; Durrant, J. R., Rate law analysis of water oxidation on a hematite surface. *Journal of the American Chemical Society* **2015,** *137* (20), 6629-6637.
5. Kafizas, A.; Ma, Y.; Pastor, E.; Pendlebury, S. R.; Mesa, C.; Francàs, L.; Le Formal, F.; Noor, N.; Ling, M.; Sotelo-Vazquez, C., Water oxidation kinetics of accumulated holes on the surface of a TiO2 photoanode: a rate law analysis. *Acs Catalysis* **2017,** *7* (7), 4896-4903.
6. Zhao, Y.; Ding, C.; Zhu, J.; Qin, W.; Tao, X.; Fan, F.; Li, R.; Li, C., A hydrogen farm strategy for scalable solar hydrogen production with particulate photocatalysts. *Angew. Chem. Int. Ed.* **2020,** *59* (24), 9653-9658.
